# Dietary Total Antioxidant Capacity and Gestational Diabetes Mellitus: A Case-Control Study

**DOI:** 10.1155/2020/5471316

**Published:** 2020-10-08

**Authors:** Elnaz Daneshzad, Hatav Tehrani, Nick Bellissimo, Leila Azadbakht

**Affiliations:** ^1^Department of Community Nutrition, School of Nutritional Science and Dietetics, Tehran University of Medical Sciences, Tehran, Iran; ^2^Department of Obstetrics and Gynecology, Isfahan University of Medical Sciences, Isfahan, Iran; ^3^School of Nutrition, Ryerson University, Toronto, Canada; ^4^Diabetes Research Center, Endocrinology and Metabolism Clinical Sciences Institute, Tehran University of Medical Sciences, Tehran, Iran; ^5^Department of Community Nutrition, School of Nutrition and Food Science, Isfahan University of Medical Science, Isfahan, Iran

## Abstract

**Background:**

Elevated oxidative stress status has been reported among pregnant women with gestational diabetes mellitus (GDM). In diabetic condition, glucose and lipid peroxidation, and alteration in antioxidant defense lead to increased free radicals. The objective of this study was to investigate the association between dietary total antioxidant capacity (DTAC) and GDM.

**Methods:**

This hospital-based case-control study was conducted in 463 pregnant women (healthy, *n* = 263; GDM, *n* = 200). Anthropometric indices, blood pressure, and biochemical analyses were measured. Dietary intake was assessed by the average of three 24-hour dietary intake records. DTAC was calculated by three indices: ferric reducing ability of plasma (FRAP), total radical-trapping antioxidant parameter (TRAP), and Trolox equivalent antioxidant capacity (TEAC). Multivariable logistic regression was performed to examine the relationship between DTAC and GDM risk in crude and adjusted models.

**Results:**

The mean age and BMI were 28.33 ± 6.23 years and 29.67 ± 4.73 kg/m^2^, respectively. Total energy, protein, and selenium intakes were significantly higher in cases than controls (*P* < 0.05). Moreover, intakes of carbohydrate, vitamins C, B6, and A, manganese, fruits, fruit juices, vegetables, legumes, and FRAP were significantly lower in cases than controls (*P* < 0.05). The risk of gestational diabetes mellitus was 85% lower among those in the highest tertile of FRAP (OR: 0.15; 95% CI: 0.08-0.29). There was no significant association between the risk of GDM and TRAP (OR: 1.62; 95% CI: 0.94-2.79) as well as TEAC (OR: 1.56; 95% CI: 0.89-2.72).

**Conclusion:**

Pregnant women who were in the highest tertile of FRAP were at lower risk of GDM. However, larger prospective studies are needed to confirm our findings.

## 1. Introduction

Gestational diabetes mellitus (GDM) is a condition in which glucose metabolism is abnormal during the second and third trimester of pregnancy [1]. Early diagnosis and treatment are important because blood glucose disturbances might lead to fetal hypoxia, fetal malformation, and respiratory distress syndrome, to name a few [2–4]. Moreover, GDM may also be associated with developing type 2 diabetes, metabolic syndrome, and cardiovascular disease later in life [5]. Some studies have also reported the presence of severe oxidative stress status in diabetes as well as GDM [6, 7]. Glucose and lipid peroxidation, an alteration in antioxidant defense and oxidative destruction of glycated proteins, can lead to an increase in the production of free radicals [8–10]. Moreover, oxidative stress increases during pregnancy due to an increase in oxygen utilization and imbalance between production and scavenging of free radicals [11, 12]. An important role of dietary components is suggested as antioxidants can inhibit the activity of free radicals and neutralize them [12]. Healthy diets can improve abnormal glucose tolerance. Dietary patterns including fruits, low-fat dairies, and vegetables are associated with reduced risk of GDM [13]. In a previous study, higher intake of fruits and vegetables was associated with lower abnormal glucose tolerance in pregnant women [14]. For example, adherence to the Mediterranean and Dietary Approaches to Stop Hypertension (DASH) diet, which is rich in fruits and vegetables, is associated with reduced risk of GDM [15].

Diets rich in antioxidants improve total antioxidant status and lead to better health [16]. Measurement of total antioxidant capacity (TAC) is useful to determine the relationship to health, and the cumulative effect of antioxidants [17] may exert additive effects among antioxidants [18]. Dietary total antioxidant capacity (DTAC) shows the cumulative antioxidant capacity of foods and beverages, specifically fruits, fruits juices, and vegetables [19]. In a recent systematic review, an inverse association between DTAC and blood glucose concentrations was observed [20] through its effects on glucose metabolism [21]. Other studies have shown an inverse association between DTAC and risk of some chronic disorders such as cancer [22, 23], development of metabolic syndrome [24], lipid profile [25], inflammation [26], and biomarkers of diabetes [27].

To the best of our knowledge, there is no study which has assessed whole dietary antioxidant capacity and GDM in Iran. Therefore, this study is aimed at investigating the association between dietary total antioxidant capacity and GDM in a case-control study.

## 2. Methods

The present study was as a hospital-based case-control study, conducted in a referral hospital and nutrition clinics in Isfahan, Iran. Pregnant women (*n* = 463; based on simple sample collection and using following formula: (*r* + 1/*r*)(SD^2^(*Z*_*β*_ + *Z*_*α*_/2)^2^/*d*^2^; using fasting blood sugar variable = 109.2; *r* = 1, *Z*_*β*_ = 0.84, and *Z*_*α*/2_ = 1.96; power of 80%) [28] with a singleton pregnancy between the ages of 22 to 44 y participated in this study. Participants were between 25 and 28 weeks of pregnancy. In this study, 200 subjects had GDM and 263 women did not have GDM. Cases were chosen from Azzahra hospital, nutrition clinics, and Shahid Beheshti hospital, and controls were chosen from outpatients who referred to Azzahra and Shahid Beheshti hospital in Isfahan. Controls were the patients who attend a hospital for treatment without staying there overnight. Informed written consent was obtained from all participants. For GDM cases, we included pregnant women with abnormal fasting glucose (FG ≥ 95 mg/dl or 1-hour postprandial glucose ≥ 140 mg/dl for the first screening in pregnancy duration) [29]. Moreover, GDM was diagnosed by medical doctors. GDM was diagnosed between the 25th and 28th week of pregnancy. The participants were the new diagnosed pregnant which did not use any insulin or medication therapy. Moreover, the controls were non-GDM pregnant who their FG or 1-hour postprandial glucose levels were normal. Every pregnant participant with GDM was matched with a non-GDM pregnant for the specific week of gestational period (between 25 and 28 weeks of pregnancy). Women who were carrying multiple fetuses or women with diabetes, cancer, cardiovascular diseases and who reported any medication or hormone therapy and incomplete daily food records or abnormal energy intake (<800 or > 4200 kcal/d) were excluded. This study has been approved by Isfahan University of Medical Sciences, Isfahan, Iran.

Participant weight was measured to the nearest 100 g on a SECA scale without shoes and wearing light clothing. Height was measured using a wall tape while barefoot. Body mass index (BMI) was calculated by weight (kg)/height (m^2^). Blood pressure was measured twice after 5 min of rest using an appropriate cuff according to arm size. The average of two blood pressure measurements was recorded as the final value. Blood samples were obtained after 12-hour overnight fast and were centrifuged within 30-45 minutes of collection for 10 minutes at 500 × g and at 4°C. Blood samples were analyzed using an autoanalyzer (Selectra 2; Vital Scientific, Spankeren, Netherlands). Triacylglycerols (TGs) were measured with glutathione peroxidase. Aspartate aminotransferase (AST), alanine aminotransferase (ALT), low-density lipoprotein cholesterol (LDL-C), high-density lipoprotein cholesterol (HDL-C), serum total cholesterol (TC), and FG were measured using commercially available enzymatic kits (Pars Azmmoun, Tehran, Iran).

Dietary intake was assessed by the average of three 24-hour dietary records (2 weekday and 1 weekend). Participants were trained by an expert dietitian to complete the dietary food record forms. Food album was used to estimate and record the correct size of food portions. Moreover, the raw and cooked coefficients were considered for all foods. The dietitian checked the forms to ensure their accuracy and followed up with participants by phone when records were incomplete. All portion sizes of food records were converted to grams using household measures of Iranian foods. The Nutritionist IV software (First Databank Division, the Hearst Corporation, San Bruno, CA, USA), modified for Iranian foods, was used to assess the dietary intake.

The quantitative value of DTAC for dietary intake was calculated by three indices: ferric reducing ability of plasma (FRAP), total radical-trapping antioxidant parameter (TRAP), and Trolox equivalent antioxidant capacity (TEAC). FRAP value was obtained using published databases developed by Halvorsen et al. [30]. TRAP and TEAC values were obtained by using previously published databases for Italian foods [31]. TAC value for food items was matched to an equivalent food in each of the databases. If each food was not directly matched in a database, a proxy estimation was used based on the following procedure: (1) the mean value of similar foods when no data were available and (2) TAC values of ingredients based on portions of processed or dish-based foods.

Dietary total antioxidant capacity for every participant was obtained by multiplying the daily intake of each selected food item by its corresponding antioxidant value per food portion and summing the final values. Antioxidants from supplements were not considered in the calculation of DTAC.

Socioeconomic information of participants was obtained by health care providers. Socioeconomic status (SES) was assessed by asking the pregnant women about their education status (academic or nonacademic), car ownership, house ownership (yes and no), and family size (≤4 and >4 persons). A score of 0.5 was given for having family members of ≤4, academic educations, and house or car ownership to each subject. If they had family members of >4, had nonacademic educations, or were not home or car owners, they were given the score of 0. Finally, the scores were summed and presented as total SES score. Physical activity was recorded in MET/d by participants. Participants were asked about smoking habits; however, all of them were nonsmokers. Moreover, participants were asked about their supplementation intakes. All participants consumed iron supplement.

Energy intake, sociodemographic, anthropometric, and biochemical indices were compared among case and control groups using the independent *t*-test for quantitative variables and chi-square test for the categorical variables. Analysis of covariance (ANCOVA) was used to assess the dietary intake of case and control group by adjusting energy intake. Multivariate logistic regression was performed to examine the relationship between DTAC and GDM risk in crude and adjusted models. A crude model was performed without adjustment, and two adjusted models were performed to control the effects of potential confounders (model 1: age and energy intake, model 2: model 1+socioeconomic status; SES score, model 3: models 1 and 2+dietary fiber intake, model 4: models 1, 2, and 3+carbohydrate and protein intake; and model 5: further adjustment for BMI, supplementation, physical activity, and fat intake). Statistical analyses were performed using the SPSS software (SPSS Inc., Chicago IL, version 16), and *P* < 0.05 was considered as statistically significant.

## 3. Results

The mean age and BMI were 28.33 ± 6.23 years and 29.67 ± 4.7 kg/m^2^, respectively.  Table 1 shows the anthropometric and biochemical indices in the case (GDM) and control (healthy) groups. The mean FG, HbA1C, and TG were higher in cases compared to controls (*P* < 0.0001).

**Figure 1:**
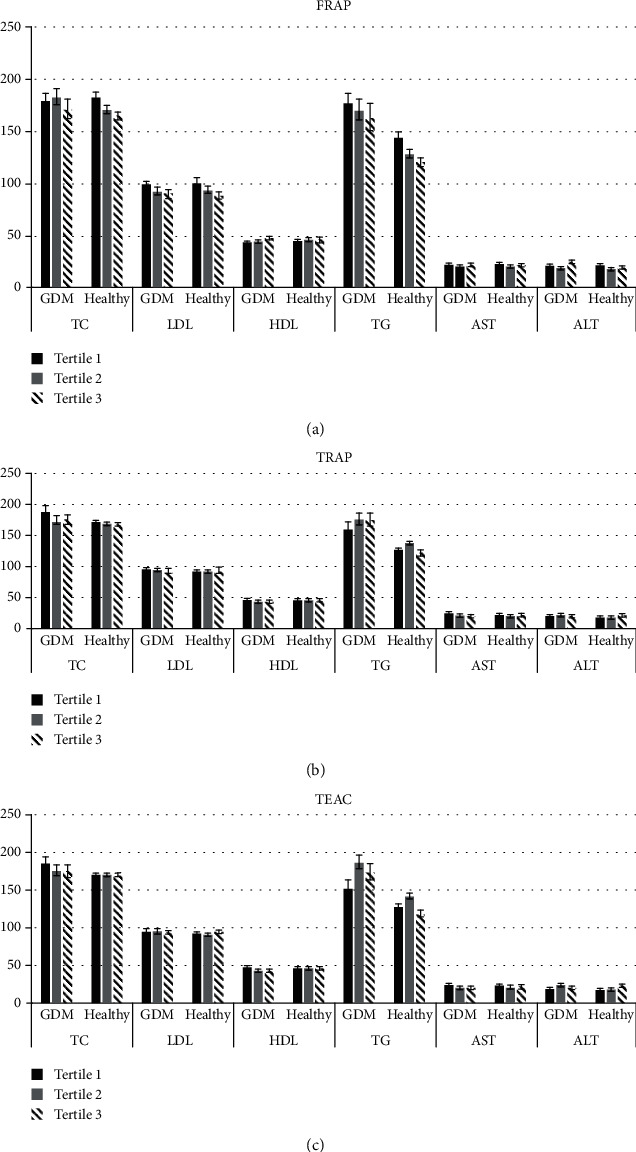
Comparison of lipid profile (mg/dl) and liver enzymes (IU/L) in GDM and healthy pregnant women among different tertiles of DTAC (presented as mean and standard error in three parts: (a–c)). (a) Comparison of lipid profile and liver enzymes in cases and controls among different tertiles of FRAP using ANCOVA. Variables have adjusted for age, energy intake, SES, number of having child, and dietary fiber intake. TG in healthy group is significant (*P* < 0.05). (b) Comparison of lipid profile and liver enzymes in cases and controls among different tertiles of TRAP using ANCOVA. Variables have adjusted for age, energy intake, SES, number of having child, and dietary fiber intake. TG and ALT in healthy group are significant (*P* < 0.05). (c) Comparison of lipid profile and liver enzymes in cases and controls among different tertiles of TEAC using ANCOVA. Variables have adjusted for age, energy intake, SES, number of having child, and dietary fiber intake. TG and ALT in healthy group and HDL in GDM group are significant (*P* < 0.05).

Figure 1 is a comparison of lipid profile and liver enzymes in GDM and healthy pregnant women across tertiles of DTAC. In Figure 1(a), TG levels were significantly lower in the highest tertile of FRAP value in healthy individuals (tertile 1: 144.02 ± 6.04; tertile 2: 129.21 ± 4.25; tertile 3: 121.24 ± 4.09); however, other variables did not indicate a statistical association. TG levels were significantly lower in the highest tertile of TRAP value in healthy individuals (tertile 1: 127.01 ± 4.16; tertile 2: 138.09 ± 4.55; tertile 3: 122.78 ± 4.51; *P* = 0.04).

Dietary intake and dietary total antioxidant capacity among the case and control groups are indicated in Table 2. Total energy, protein, and selenium intakes were significantly higher in cases than controls (*P* < 0.05). Moreover, intakes of carbohydrate, vitamins C, B6, and A, manganese, fruits, fruit juices, vegetables, legumes, and FRAP value were significantly lower in cases than controls (*P* < 0.05). There was no significant difference for TRAP and TEAC values between cases and controls (*P* > 0.05).

Multivariate odds ratios for GDM across tertiles of DTAC are reported in Table 3 in crude and adjusted models. The risk of gestational diabetes mellitus was 85% lower among those in the highest tertile of FRAP. There was no significant association between TEAC and TRAP with the risk of GDM.

## 4. Discussion

In this case-control study, mean FRAP and vitamin C were lower in GDM cases compared with a healthy control group. Based on these results, dietary total antioxidant capacity may reduce abnormal blood glucose level in pregnant women. Although there was no significant association between GDM and tertiles of TRAP and TEAC, there was a significant inverse association between GDM and FRAP. To the best of our knowledge, this is the first study that has investigated the association between GDM and DTAC.

According to several studies in healthy adults, adjusted DTAC had an inverse association with fasting blood glucose and insulin levels [32, 33]. In previous studies, the food frequency questionnaire was used to assess dietary intake. In addition, age, gender, and energy intake were considered as potential confounders [24, 32–34]. In agreement with our study, a case-control study of 80 diabetic patients and 37 controls found lower DTAC status in diabetic patients [35].

Consistent with our study, another case-control study found that prediabetes individuals have significantly lower mean DTAC than the control group [28]. Participants who were in the highest quartile of DTAC were less likely to experience elevated blood glucose concentrations after, adjusting BMI, dietary fiber, fat, and energy intake [28]. A cross-sectional study has also founded that higher DTAC is associated with lower levels of diabetes biomarkers in healthy prediabetic and diabetic individuals [27]. In our study, the significant association between DTAC and GDM persisted on even after adjusting potential confounders such as age and energy intake.

Fruits are rich source of antioxidants as well as vitamins, minerals, terpenes, lignans, polyphenols, and flavonoids. Positive associations have been observed between plasma TAC and fruit, moreover between plasma TAC and vegetable intake [36]. Fruits have low energy density and low glycemic load; moreover, their antioxidant capacity leads to a diminishment of oxidative stress, which is why they are beneficial for increasing insulin sensitivity and pancreatic *β* cell function [37–39]. Whole grains have high antioxidant capacity, in addition, contribute to lower absorption of glucose [40]. Fruits, vegetables, and whole grains as sources of dietary fiber have beneficial effects on diabetes-related biomarkers [41]. In the present study, the association between FRAP value and GDM remained significant even after adjusting dietary fiber.

Pancreatic *β* cells are vulnerable to the effects of oxidative stress due to lack of enzymatic antioxidants; therefore, oxidative stress, which damages mitochondria, reduces insulin secretion and increases blood glucose [42, 43]. A decline in antioxidant defense and increase in radical oxygen species can lead to damage of cellular organelles and enzymes, increase proteins and lipids peroxidation, and develop insulin resistance [44, 45]. Therefore, the adequate intake of antioxidants may be important for the maintenance of glucose homeostasis.

Abnormal glucose metabolism and increased glycemia condition can develop complications and health disturbances which lead to fetal hypoxia, fetal malformation, and respiratory distress syndrome; therefore, early diagnosis and all kind of treatment approaches such as improving lifestyle, diet therapy, and healthy food choices are important to control the blood glucose levels during pregnancy and reduce the probability of developing type 2 diabetes, metabolic syndrome, and cardiovascular disease later in life [2–4]. Increased weight during pregnancy develops insulin resistance. Moreover, insulin resistance and obesity increase oxidative stress in women with GDM. Therefore, consuming high antioxidant foods or nutrients and increased dietary antioxidant capacity may contribute to delay or prevent the initiation or progression of GDM [3–5].

In the present study, TG levels were significantly lower in the highest tertile of FRAP value in healthy individuals. Also, TG levels were significantly lower in the highest tertile of TRAP value in healthy individuals. Inconsistent results are presented in different studies. Bahadoran et al. and Georgoulis et al. [46, 47] failed to find any significant association between DTAC and TG. In line with our study, a study showed an inverse association between DTAC and TG; however, DTAC was measured by TEAC [24]. Moreover, Kim et al. revealed an inverse association between DTAC quartiles and TG [25]. Dietary antioxidants contribute to reducing cholesterol absorption and suppressing cholesterol synthesis and intermediate role of insulin resistance [48].

In the result description, we should consider that DTAC was based on dietary antioxidants and did not consider antioxidant bioavailability or metabolism. Moreover, three issues for assessing DTAC are developed as rapid *in vitro* methods and are different in basic calculation. In fact, these three methods differ on the chemical background and nature of redox system. TRAP measures the chain-breaking antioxidant capacity and involves the transfer of hydrogen atoms; FRAP measures the iron-reducing power of antioxidants, and TEAC measures the antioxidant capacity to quench free radicals and involve electron transfer reactions. These three databases include different numbers of foods such as vegetables, fruits, nuts, and dried fruits.

The present study has several limitations. The case-control design is the main limitation due to its inability to support cause and effect relationships. Also, one of the well-known limitations is the use of a 24-hour dietary record to assess dietary intake which leads to reproducibility and recall bias. Moreover, the findings of this study might not be generalizable to other populations without GDM. It might not be useful for FRAP assay for assessing total antioxidant capacity levels and its association with GDM. As there is no database for the content of Iranian food antioxidant, using a database for Italian food is another limitation of the study. Finally, although we assessed the supplement intake and adjusted their residual effect in the analysis, we just calculated the dietary antioxidant capacity and did not consider the supplement intake for calculation of antioxidant capacity. On the other hand, the strengths of the present study are adequate sample size and adjustment for several potential confounders. Additionally, despite there is no valuable data about DTAC of Iranian foods, we included all three assays for calculating DTAC.

The present study is aimed at investigating the association between dietary total antioxidant capacity and GDM. Based on these results, pregnant women who were in the highest tertile of FRAP were at lower risk for GDM. As it is well-known that healthy eating patterns have important applications to the prevention and treatment of chronic diseases, dietary antioxidant consumption may be have beneficial effects in this regard and may be related to clinical practices. Future clinical trials that consider various confounders and larger prospective studies with other dietary assessment tools such as dietary history questionnaires or food frequency questionnaires are needed to find the exact relationships.

## Figures and Tables

**Table 1:** Anthropometric and biochemical indices among the cases^∗^ and control groups.

Variables	Groups, mean ± SD		*P* value
	Case (*n* = 200)	Control (*n* = 263)	
Age, y	29.27 ± 5.96	27.61 ± 6.34	0.004
Weight, kg	74.45 ± 13.10	74.53 ± 12.97	0.943
BMI, kg/m^2^	29.67 ± 4.88	29.67 ± 4.62	0.994
Waist circumference, cm	96.17 ± 15.03	98.11 ± 17.73	0.215
Physical activity, MET/h	9.61 ± 0.48	9.59 ± 0.46	0.749
SBP, mmHg	120.15 ± 15.61	120.26 ± 17.72	0.464
DBP, mmHg	74.92 ± 9.01	76.11 ± 8.50	0.130
FG, mg/dl	148.00 ± 48.92	78.20 ± 10.94	<0.0001
HbA1C (%)	7.81 ± 1.68	5.58 ± 1.16	<0.0001
TC, mg/dl	179.07 ± 62.38	170.90 ± 38.14	0.083
TG, mg/dl	172.14 ± 86.32	128.87 ± 40.69	<0.0001
LDL-C, mg/dl	95.52 ± 30.03	93.53 ± 34.33	0.516
HDL-C, mg/dl	45.60 ± 9.43	47.25 ± 9.17	0.059
ALT, IU/L	21.99 ± 14.74	20.11 ± 10.57	0.112
AST, IU/L	22.57 ± 15.62	22.60 ± 12.98	0.977
SES score	1.63 ± 0.90	1.45 ± 0.80	0.027
Multivitamin intake; *n* (%)	39 (19.5)	50 (19.2)	0.895

^∗^Case: women with gestational diabetes mellitus. BMI: body mass index; SBP: systolic blood pressure; DBP: diastolic blood pressure; FG: fasting glucose; HbA1c: hemoglobin A1C; TC: total cholesterol; TG: triglyceride; LDL-C: low-density lipoprotein cholesterol; HDL-C: high-density lipoprotein cholesterol; ALT: alanine aminotransferase; AST: aspartate aminotransferase; SES: socioeconomic status.

**Table 2:** Dietary intake and dietary total antioxidant capacity among the cases^∗^ and control groups.

Dietary intake	Groups		*P* value^∞^
	Case (*n* = 200)	Control (*n* = 263)	
Total energy intake, kcal	1970.32 ± 500.06	1861.89 ± 612.86	0.042
Carbohydrate, g	320.87 ± 2.79	329.09 ± 2.43	0.027
Fat, g	46.27 ± 1.19	45.03 ± 1.04	0.433
Protein, g	67.57 ± 1.16	62.71 ± 1.01	0.002
Fiber, g	26.51 ± 0.58	26.60 ± 0.50	0.906
Vitamin D, *μ*g/d	1.30 ± 0.09	1.17 ± 0.07	0.308
Vitamin C, mg/d	265.18 ± 6.55	307.41 ± 5.72	<0.0001
Vitamin E, IU/d	7.30 ± 0.35	7.35 ± 0.31	0.913
Vitamin A, IU/d	1385.87 ± 58.80	1572.87 ± 51.35	0.017
Calcium, mg/d	1248.56 ± 31.74	1270.27 ± 27.71	0.607
Zinc, mg/d	8.89 ± 0.14	9.13 ± 0.13	0.228
Magnesium, mg/d	369.21 ± 5.97	371.50 ± 5.21	0.773
Vitamin B_2_, IU/d	2.25 ± 0.04	2.31 ± 0.03	0.227
Vitamin B_6_, mg/d	1.71 ± 0.03	1.82 ± 0.02	0.008
Selenium, *μ*g/d	0.07 ± 0.002	0.06 ± 0.002	0.002
Iron, mg/d	12.51 ± 0.19	12.53 ± 0.17	0.919
Manganese, mg/d	4.12 ± 0.10	4.41 ± 0.08	0.028
Fruits, g	584.36 ± 15.84	663.781 ± 13.91	<0.0001
Fruit juices, g	5.44 ± 1.10	8.50 ± 0.96	0.038
Vegetables, g	449.54 ± 15.64	500.39 ± 13.73	0.015
Nuts, g	11.45 ± 1.29	14.50 ± 1.13	0.077
Egg, g	9.10 ± 0.56	10.26 ± 0.72	0.125
Rice, g	317.27 ± 12.05	328.95 ± 10.52	0.467
Legumes, g	22.14 ± 1.23	28.73 ± 1.08	<0.0001
Grains, g	402.83 ± 11.93	402.90 ± 10.41	0.997
Red meat, g	8.12 ± 0.37	8.57 ± 0.32	0.361
Fish and chicken, g	14.73 ± 0.74	17.51 ± 0.64	0.005
Processed meat, g	1.78 ± 0.34	5.18 ± 0.30	<0.0001
TRAP, mmol/d	8.34 ± 0.26	8.60 ± 0.23	0.462
FRAP, mmol/d	11.13 ± 0.28	12.88 ± 0.25	< 0.0001
TEAC, mmol/d	7.18 ± 0.20	7.45 ± 0.17	0.311

^∗^Case: women with gestational diabetes mellitus. ^∞^All food values presented as mean and standard error using ANCOVA test which adjusted for energy intake. Total energy intake presented as mean and standard deviation ANOVA. TRAP: total radical-trapping antioxidant parameter; FRAP: ferric reducing ability of plasma; TEAC: Trolox equivalent antioxidant capacity.

**Table 3:** Odd ratio for gestational diabetes mellitus in different tertiles of dietary total antioxidant capacity indices.

	1	2	3	*P* trend^∞^
	Tertiles of TRAP			
Crude model	1	1.17 (1.08-2.69)	1.38 (0.87-2.19)	0.167
Model 1	1	1.60 (0.99-2.56)	1.25 (0.76-2.06)	0.381
Model 2	1	1.63 (1.00-2.63)	1.38 (0.83-2.30)	0.211
Model 3	1	1.61 (0.99-2.61)	1.36 (0.81-2.27)	0.237
Model 4	1	1.53 (0.94-2.51)	1.45 (0.85-2.45)	0.156
Model 5	1	1.49 (0.91-2.45)	1.62 (0.94-2.79)	0.072
	Tertiles of FRAP			
Crude model	1	0.44 (0.27-0.70)	0.26 (0.16-0.42)	<0.0001
Model 1	1	0.40 (0.25-0.66)	0.23 (0.13-0.38)	<0.0001
Model 2	1	0.43 (0.26-0.71)	0.23 (0.14-0.40)	<0.0001
Model 3	1	0.36 (0.21-0.61)	0.16 (0.09-0.30)	<0.0001
Model 4	1	0.33 (0.19-0.57)	0.15 (0.08-0.29)	<0.0001
Model 5	1	0.31 (0.17-0.53)	0.15 (0.08-0.29)	<0.0001
	Tertiles of TEAC			
Crude model	1	1.50 (0.95-2.37)	1.34 (0.85-2.10)	0.201
Model 1	1	1.35 (0.84-2.18)	1.18 (0.72-1.95)	0.509
Model 2	1	1.52 (0.93-2.48)	1.40 (0.83-2.36)	0.204
Model 3	1	1.52 (0.93-2.48)	1.36 (0.80-2.31)	0.243
Model 4	1	1.37 (0.83-2.26)	1.37 (0.80-2.34)	0.239
Model 5	1	1.32 (0.80-2.19)	1.56 (0.89-2.72)	0.113

^∞^ *P* trends calculated by binary logistic regression. Model 1 has adjusted for age and energy intake. Model 2 is model 1+SES and number of offspring. Model 3 is models 1 and 2+dietary fiber intake. Model 4 is models 1, 2, and 3+carbohydrate and protein intake. Model 5 is the further adjustment for BMI, supplementation, physical activity, and fat intake. TRAP: total radical-trapping antioxidant parameter; FRAP: ferric reducing ability of plasma; TEAC: Trolox equivalent antioxidant capacity.

## Data Availability

The data used to support the findings of this study are available from the corresponding author upon request.
